# Clinical relevance of deep learning models in predicting the onset timing of cancer pain exacerbation

**DOI:** 10.1038/s41598-023-37742-5

**Published:** 2023-07-17

**Authors:** Yeong Hak Bang, Yoon Ho Choi, Mincheol Park, Soo-Yong Shin, Seok Jin Kim

**Affiliations:** 1 Department of Digital Health, Samsung Advanced Institute for Health Sciences and Technology, Sungkyunkwan University, 81 Irwon-Ro, Gangnam-Gu, Seoul, Korea; 2 Department of Oncology, Asan Medical Center, University of Ulsan College of Medicine, Seoul, Korea; 3 Center for Artificial Intelligence, Korea Institute of Science and Technology, Seoul, Korea; 4 Division of Hematology and Oncology, Department of Medicine, Samsung Medical Center, Sungkyunkwan University School of Medicine, 81 Irwon-Ro, Gangnam-Gu, Seoul, Korea

**Keywords:** Palliative care, Pain

## Abstract

Cancer pain is a challenging clinical problem that is encountered in the management of cancer pain. We aimed to investigate the clinical relevance of deep learning models that predict the onset of cancer pain exacerbation in hospitalized patients. We defined cancer pain exacerbation (CPE) as the pain with a numerical rating scale (NRS) score of ≥ 4. We investigated the performance of the deep learning models using the Matthews correlation coefficient (MCC) with different input lengths and time binning. All the pain records were obtained from the electronic medical records of the hematology-oncology wards in a Samsung Medical Center between July 2016 and February 2020. The model was externally validated using the holdout method with 20% of the datasets. The most common type of cancer was lung cancer (n = 745, 21.7%), and the median CPE per day was 1.01. The NRS pain records showed circadian patterns that correlated with NRS pain patterns of the previous days. The correlation of the NRS scores showed a positive association with the closeness of the NRS pattern of the day with forecast date and size of time binning. The long short-term memory-based model exhibited a good performance by demonstrating 9 times the best performance and 8 times the second-best performance among 21 different settings. The best performance was achieved with 120 h input and 12 h bin lengths (MCC: 0.4927). Our study demonstrated the possibility of predicting CPE using deep learning models, thereby suggesting that preemptive cancer pain management using deep learning could potentially improve patients’ daily life.

## Introduction

Cancer pain is common in patients, particularly during the advanced stages of the disease, when the prevalence is estimated to be more than 40%. This contributes to poor physical and emotional state of the patients^1, 2^. Prolonged survival, followed by advancement in diagnosis and treatment of cancer, results in an increase in the number of patients experiencing persistent pain^3, 4^. This trend has also been documented in hematology patients at the time of diagnosis, during therapy, and in the last month of their life^5, 6^. As per the estimates of GLOBOCAN 2020, the incidences of cancer is increasing and will be more than 19 million^7^. Thus, cancer-related pain would be a major issue in global healthcare systems.

Acute exacerbation of cancer pain is a challenging clinical problem in managing cancer pain, negatively impacting the patient’s daily life^8–11^ Some guidelines suggest that the occurrence of three to four cancer pain exacerbation (CPE) episodes per day is acceptable^12^. However, a less frequent CPE could also affect patients’ daily quality of life. In addition, the interval between pain onset time and drug effect time could worsen the patients’ quality of life. In particular, hospitalized patients can only avail short-acting opioids upon informing the nurse. Thereafter, the nurse must inform the doctor for a decision. The patients would likely be left in severe pain during this processing time. From this point of view, accurate prediction of CPE could alleviate the frequency and interval of CPE and improve patients’ daily life well-being.

Recently, the time series model based on a deep learning algorithm has gained popularity based on its remarkable performance^13, 14^. Cancer pain may reflect the status of cancer invasiveness, exposure to cancer treatment, pharmacodynamics of the opioids, patient’s lifestyle, and the health care system of the hospital, including the management of doctors and nurses^1^. Therefore, we hypothesized that the exacerbation of cancer pain is repeated according to the patient’s previous patterns and could thus, be predictable. In this study, we aimed to investigate the clinical relevance of deep learning models that predict the time of breakthrough pain onset in cancer patients.

## Patients and methods

### Patients and data collection

**Figure 1:**
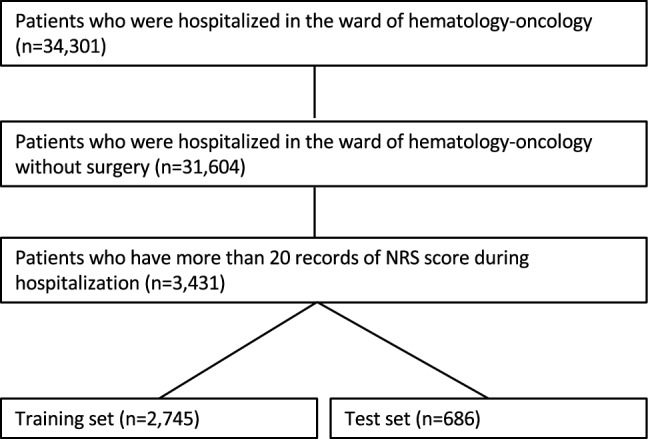
The study cohort.

This single-center retrospective study aimed to evaluate the efficacy of deep learning model in predicting the onset of CPE in cancer patients. This study was conducted in accordance with the Declaration of Helsinki and was approved by the institutional review board (IRB) at the Samsung Medical center (approval number 2020–09-073). The Samsung Medical Center IRB waived the need for informed consent because of the retrospective nature of this study. All pain records pertaining to 34,304 patients were retrospectively collected who were admitted to the department of hematology and oncology of the Samsung Medical center in Korea between July 2016 and February 2020. Clinical data were obtained from the medical records using de-identified clinical data warehouse^15^. Of all the patients, we excluded 2,697 patients who underwent surgery during hospitalization and 28,173 patients with less than 20 non-zero numerical rating scale (NRS) score records. The selected 3,431 patients included pain log data for 4,870 admissions, split into the 80–20% training/test (2,745/686 patients with 3,896/974 admissions) set (Fig. 1).

### Pre-processing

**Figure 2:**
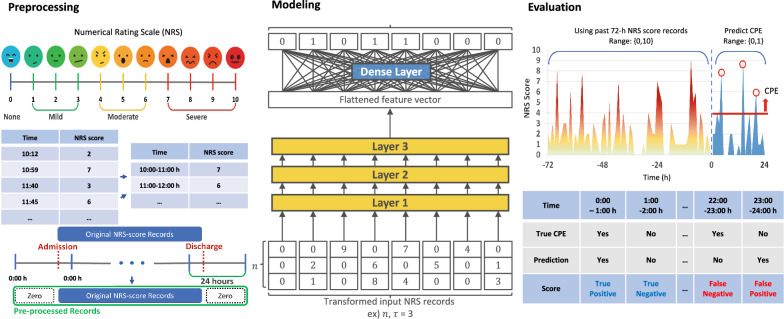
Data processing, modeling, and evaluation schemes.

Nurse rounding occurs every day at 5:00 h, 13:00 h, and 21:00 h. During nurse rounding, nurses usually record the patient’s self-reported pain scores using NRS scales with an ordinal range from 0 (no pain) to 10 (severe and unbearable pain)^16^. The patients could also ask the nurse for management at other times in the case of sudden pain, Herein, the nurse records the additional pain scores and notifies the doctors. As there was no quantitative consensus on the definition of CPE, we defined CPE as an NRS score of 4, a commonly used indication of opioid intervention in cancer pain management guideline[2]. Pre-processing consists of binning and transformation steps as mentioned below. Figure 2 explains the entire process.

#### Binning

Originally, the pain scores were recorded according to the time in minutes. However, considering the highly sparse signal of the data, we binned the entire NRS record in arbitrary $\tau$-length time-bins. If multiple records were within the same bin, the highest NRS score was considered as the record for that period.

#### Transformation

CPE could be different among patients (inter-patient) and could also be different in the specific situation of each patient (intra-patient). Intra-patient pain pattern refers to a patient's unique pain pattern depending on the patient’s medical status. Inter-patient pain pattern refers to the pattern of medical practice, including rounding time and clinician’s management. All pain records were processed in 24-h increments to reflect these characteristics. Zero padding was used to set the start and end of all pain records to 0:00 h. Recordings at time points where there were no NRS observations in the episode, the patient was considered pain-free and were imputed to zero. Accordingly, pain records for $n$ days with $\tau$-length time-bins were transformed from a shape of $1\times ((24/\tau )\times n)$ vector form to a ($24/\tau )\times n$ matrix form.

### Modeling

We explored six deep learning-based time series forecasting architectures for prediction of CPE according to the various input length and time-bin size $(\tau )$ of pain records. Time-bin size ($\tau )$ was investigated by grid search for the divisors of 24, suited for transformation, excluding 24, considering the clinical application ($\tau \in$ {1, 2, 3, 4, 6, 8, 12} h). The comparison was conducted for recurrent neural network (RNN)^17^, long short-term memory (LSTM)^18^, gated recurrent unit (GRU)^19^, bidirectional long short-term memory (Bi-LSTM)^20^, hybrid of the convolutional neural network, long short-term memory (CNN-LSTM)^21^, and transformer^22^. Each prediction model of CPE was implemented according to its respective basic recipe, and the models were constructed with a non-autoregressive prediction structure followed by a dense layer after stacking three basic blocks (Fig. 2). To make a fair comparison, the number of hyperparameters between all models was set to minimize their differences (within 3,000 parameters). The model was trained for 300 epochs with a batch size of 100. A balanced cross entropy loss was ensured and the system was optimized by stochastic weight averaging^23^ with an initial learning rate of 1e-4, start averaging of 5, and the average period of 1. Our model was programmed in Python 3.7, Tensorflow 2.4.1, and experimented using NVIDIA GeForce RTX 2080.

### Evaluation

The prediction of CPE could be regarded as a binary classification of independents (0 s and 1 s) over the next 24 h (Fig. 2), and the number of trues depends on the time-bin size ($\tau )$, which shows a class imbalance with a dominant. Therefore, we evaluated the performance of the model based on the Matthews correlation coefficient (MCC), a reliable statistical proportion that produced a high score proportional to the size of the positive and negative elements in the dataset. This is possible only if the predictions yield good results in all four categories of confusion matrix (true positive [TP], false positive [FP], true negative [TN], and false negative [FN])^24^. The range of MCC values is [− 1, 1], where − 1 indicates the opposite of prediction and trues, and + 1 indicates the correct. In addition to MCC, the performance of the model was also evaluated with area under the receiver operating characteristic curve (AUROC) and area under the precision-recall curve (AUPRC).

### Ethics approval and patient consent statement

The ethical review board of Samsung Medical Center approved the study protocol. As per the regulations in Korea, the review board waived the requirement for informed consent for this study as it was a retrospective analysis.

## Results

### Baseline characteristics of patients

The baseline characteristics of 3,431 patients and 4,870 admissions are shown in Supplementary Table 1. The median age was 58 years (range, 15–89), and 2,047 (59.7%) were male. The most common type of cancer was lung cancer (n = 745, 21.7%), followed by lymphoma (n = 491, 14.3%). The median hospitalization duration was 14.96 days (range, 5.46–195.40), and patients with aplastic anemia were hospitalized with the longest duration (29.50 days [range, 7.79–93.10]). The median frequency of records of NRS and CPE per day was 2.74 (range, 0.17–12.22) and 1.01 (range, 0–1.31), respectively. Patients with head and neck cancer present CPE most frequently (1.57 per day [range, 0–7.85]).

### Characteristics of pain records

**Figure 3:**
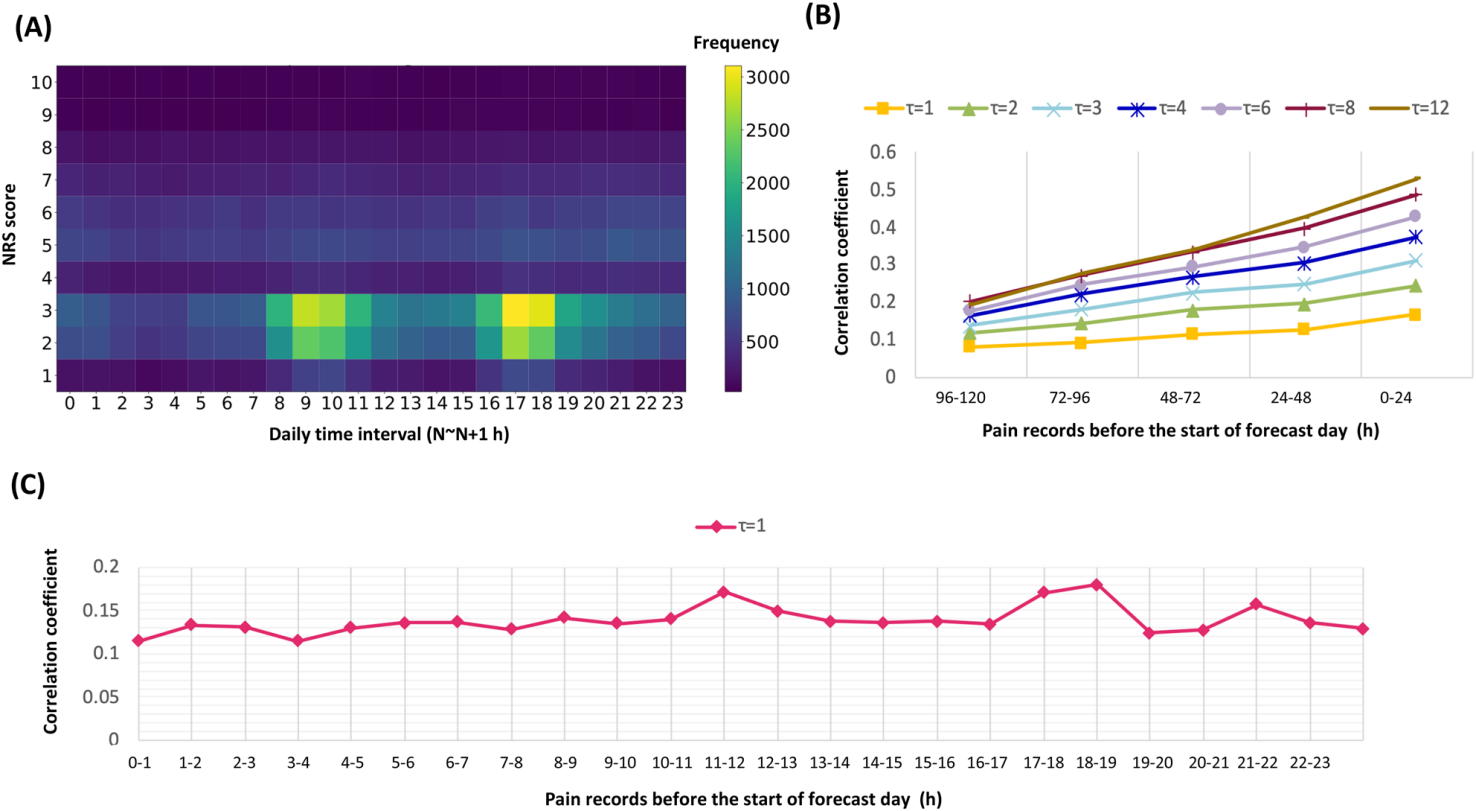
Characteristics of pain records. (**A**) Heatmap of the daily pain pattern of all patients. (**B**) Correlation analysis of the NRS scores between the forecast date and prior records. We used the Pearson correlation coefficient to evaluate the correlation between pain records according to the days. (**C**) Correlation analysis of the time-matched NRS scores between the forecast date and the previous day.

Pain scores were mostly recorded at 8:00–10:00 h, and 16:00–18:00 h, which were the regular rounding times at the ward (Fig. 3A). Among the 1-h time binned records (n = 1,311,240), 78,376 (6.0%) was CPE, while more frequent CPE were noted with larger time binned records, showing 44.5% (n = 48,663) of CPE in the 12-h time binned record (n = 109,270) (Supplementary Table 2). Figure 3B described the correlation between daily pain records of forecast days and previous days. The Pearson coefficient scores increased closer to the forecast period with the increase in time binning. In the setting of an hour time binned records, the NRS record in the interval between 96 and 120 h before forecast day showed a coefficient of 0.08. The score was improved closer to forecast date, which showed a coefficient of 0.20 in records that were made 24 h before the forecast date. The Pearson coefficient scores increased with larger time bin records, presenting a coefficient of 0.53 in 12-h time binned NRS records of the previous day. The correlation analysis of matched time NRS scores between the forecast day and a day prior to the forecast period indicated that the interval between 11:00 h and 12:00 h (Coefficient: 0.17) and 18:00 to 19:00 h showed relatively high correlations (Coefficient: 0.18) compared to others (Fig. 3C).

### Comparison of performance

Table 1 presents the MCC values for each deep learning model across the various range of input lengths and time-bins. In all settings, there was no significant difference in CPE prediction performance according to the structural variation of each model. The average difference between the highest and lowest performance models for each experimental condition was 0.0182, and the standard deviation was 0.0087. Nevertheless, the LSTM-based model showed a good performance. It displayed the best performance on nine occasions, while the second-best performance was recorded eight times among the 21 experiment settings. The model based on GRU, a simplified version of LSTM, also showed good performance. Herein, eight best performances and seven second-best performances were recorded. Through this exploratory investigation, we selected the LSTM block as the backbone structure of the representative CPE prediction mode. Meanwhile, all deep learning-based models were consistently better than the MCC of the base model that makes predictions with average scores of same time intervals of previous days in the window period.

**Table 1:** Performance comparisons for various models according to input and time-bin length.

Input length (h)	Model	Time-bin length $\tau$						
		1	2	3	4	6	8	12
24	Transformer	0.1616	0.2319	0.2813	0.3279	0.3735	0.4059	0.4174
	CNN + LSTM	0.1595	0.2265	0.2762	0.2988	0.3708	0.3791	0.4178
	Bi-LSTM	0.1666	0.2330	0.2807	0.3325	**0.3744**	0.4062	**0.4180**
	LSTM	**0.1721**	**0.2417**	0.2900	**0.3365**	0.3718	0.4074	0.4179
	GRU	0.1696	0.2406	**0.2912**	0.3351	0.3705	0.4055	0.4171
	RNN	0.1669	0.2328	0.2832	0.3320	0.3703	**0.4084**	0.4167
	Base model*	0.1203	0.1851	0.2413	0.2920	0.3457	0.3867	0.4058
72	Transformer	0.1764	0.2598	0.3116	0.3589	0.4100	0.4476	0.4685
	CNN + LSTM	0.1872	0.2562	0.3116	0.3617	0.4118	0.4529	0.4485
	Bi-LSTM	0.1805	0.2501	0.3016	0.3514	0.4079	0.4523	0.4745
	LSTM	0.1889	0.2650	**0.3238**	**0.3722**	**0.4233**	**0.4624**	0.4712
	GRU	**0.1938**	**0.2735**	0.3170	0.3710	0.4223	0.4618	**0.4796**
	RNN	0.1829	0.2537	0.3149	0.3636	0.4136	0.4478	0.4717
	Base model*	0.1218	0.2019	0.2565	0.3194	0.3756	0.4133	0.4343
120	Transformer	0.1761	0.2623	0.3213	0.3688	**0.4315**	0.4620	0.4879
	CNN + LSTM	0.1929	0.2658	0.3207	0.3717	0.4218	0.4615	0.4900
	Bi-LSTM	0.1815	0.2496	0.3068	0.3581	0.4142	0.4680	0.4830
	LSTM	0.1861	0.2635	**0.3231**	0.3771	0.4278	0.4730	**0.4927**
	GRU	**0.1963**	**0.2717**	0.3196	**0.3791**	0.4267	**0.4748**	0.4923
	RNN	0.1788	0.2537	0.3097	0.3734	0.4206	0.4674	0.4924
	Base model*	0.1174	0.1938	0.2591	0.3211	0.3770	0.4125	0.4297

*Base model: model that makes predictions with average scores of every hour of prior days.

The performance was evaluated based on the Matthew correlation coefficient (MCC).

Bold scores indicate the best result per set, and underlined scores mean the second performance of each experiment setting.

**Figure 4:**
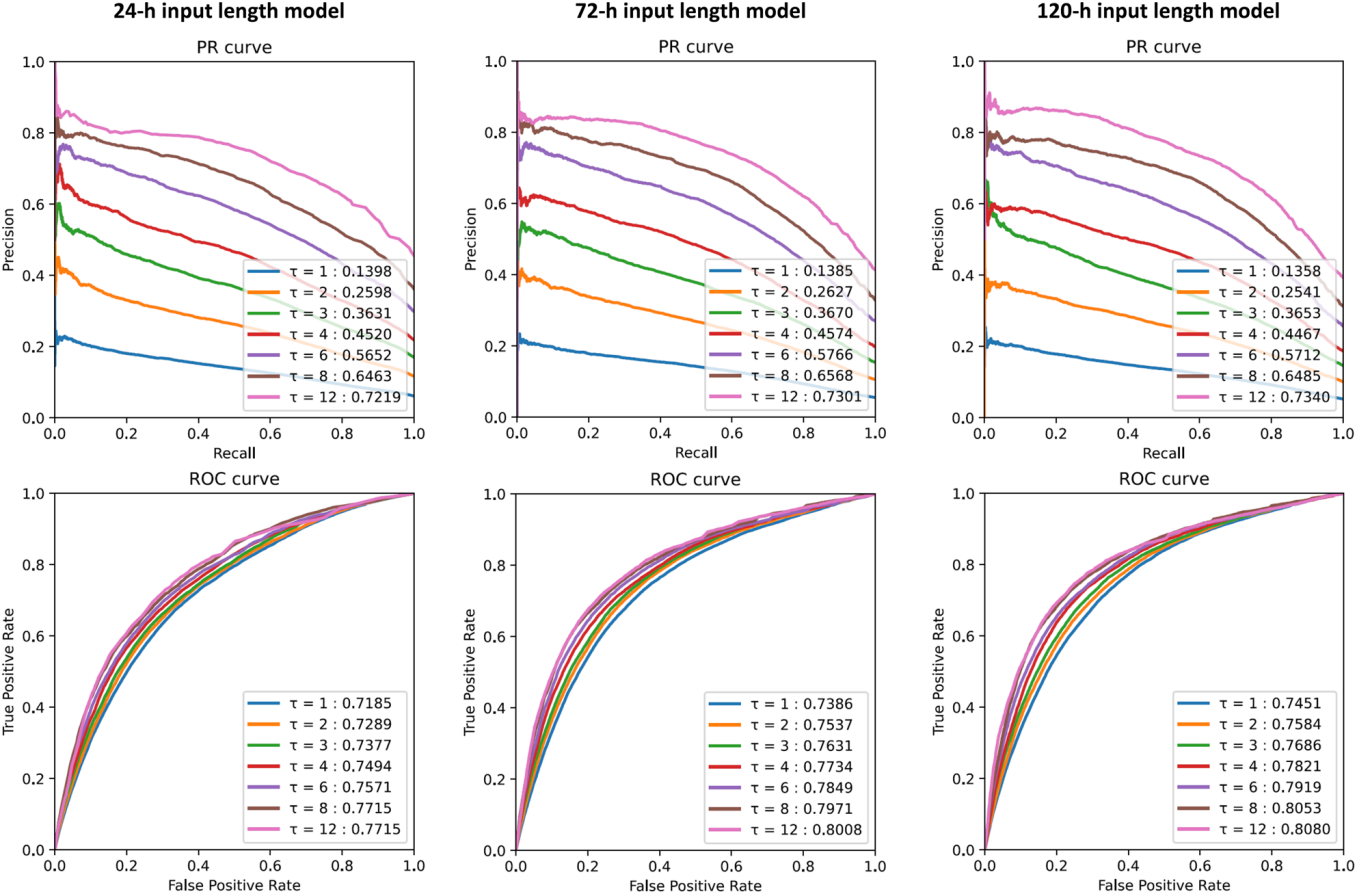
AUROC and AUPRC of LSTM-based model with different sizes of time binning and input lengths. $\tau$: time bin size.

To investigate the efficacy of transformation, we investigated the performance of LSTM-based model input NRS before transformations (Supplementary Table 3). In the setting of 24 h input and 1 h time bin, the LSTM based model using transformed data showed an MCC of 0.1721, which was better than the performance input with original NRS records (MCC: 0.1686). The model performance was consistently improved after transformation in the various input and time-bin lengths. Also, the performance was not significantly improved with a larger input length. In particular, the model input of 120 h of NRS records did not show a significantly better performance than the model input 72 h of NRS records in various time bin length settings. Instead, the performance was greatly improved with larger-sized time binning. In this study, the best performance was an MCC of 0.4927 derived from LSTM-based model using 120 h of input length and 12 h time bin set, showing AUROC of 0.8080 and AUPRC of 0.7340 (Fig. 4). This model showed best performance in patients with aplastic anemia (MCC: 0.663), followed by head and neck cancer (MCC: 0.594) (Supplementary Table 4).

### Cases of prediction for onset timing of breakthrough pain

**Figure 5:**
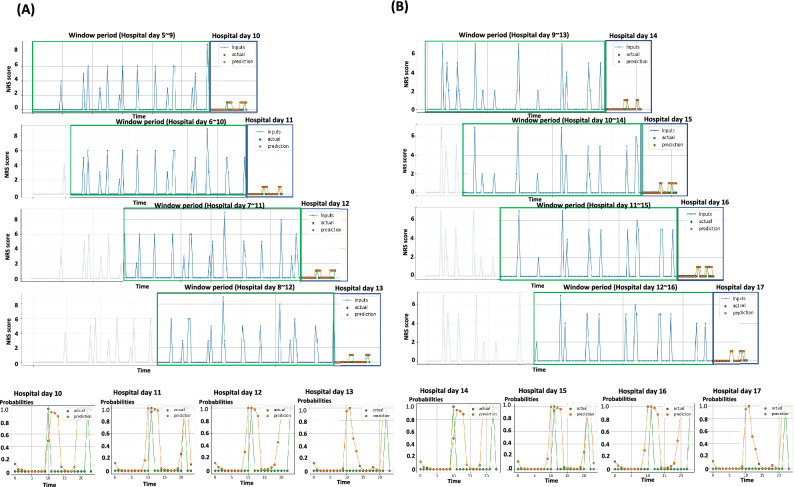
Representative cases for predicting the onset time of cancer pain exacerbation using serial pain records derived from the patients. (**A**) The case of a patient with bladder cancer who complained of back pain. Blue dots and lines indicated NRS pain scores during the window period, and yellow dots and lines were the prediction results about the presence of CPE, derived from LSTM-based models with a 5-day input length and 1-h time binned data setting. The green dots and lines indicated the real-world records about the existence of CPE according to the times. Yellow dots and lines in the black-colored box showed the probabilities of CPE on the forecast day. (**B**) The case of a patient with aplastic anemia who underwent allo-PBSCT.

Figure 5-A depicts the case of a 76-year-old male patient with bladder cancer and retroperitoneal lymph node metastasis who was admitted for supportive care. The patient showed good performance (MCC: 0.3452, AUROC: 0.9051, AUPRC: 0.2067) as pert the LSTM-based model with an hour time binning and a 5-day input data during hospitalization. He complained of back pain and was administered 12.5 mcg/h of fentanyl patch and received 5 mg of morphine (intravenous) when they presented breakthrough pain. On the tenth day. patients complained of back pain at 10:00 h and 22:00 h, and the LSTM-based model predicted the onset time of breakthrough pain between 11:00–13:00 h and 18:00–23:00 h. This value was consistently close to the actual patients’ complaints after tenth day of hospitalization. In addition, the LSTM-based model was tested in patients with hematologic malignancy. Figure 5B shows the case of a 53-year-old female patient with aplastic anemia who displayed a relatively accurate prediction during hospitalization (MCC: 0.1843, AUROC: 0.2015, and AUPRC: 0.1767). She was admitted for allogeneic peripheral blood stem cell transplant (allo-PBSCT). The patient received conditioning chemotherapy on the first hospital day, followed by allo-PBSCT, 7 days later. On 14th hospital day, the patient complained of fibromyalgia at 13:00 h and 21:00 h, and the LSTM-based model predicted onset times at 11:00 h and 20:00 h.

Supplementary Figure 1A shows other cases capturing breakthrough pain incorrectly. The patient with renal cell carcinoma, bone and pleural metastasis was administered for pleural effusion and back pain management. The LSTM-based model incorrectly predicted CPE during hospitalization (MCC: 0.0417, AUROC: 0.5843 and AUPRC: 0.1767). He complained of back pain frequently on the fifth and sixth hospital days, the day after the second thoracentesis. On the seventh hospital day, the model predicted CPE as a similar pattern to the sixth day in the hospital. However, this patient complained of CPE less often after doctors escalated the dose from 5 mg of long-acting oxycodone to 10 mg of long-acting oxycodone on the sixth hospital day. Supplementary Figure 1B depicts a patient with stomach cancer with peritoneal seeding. This patient complained of severe abdominal pain and was hospitalized for management of afferent loop syndrome. He underwent percutaneous transhepatic biliary drainage (PTBD) and L-tube insertion in the ER. However, the PTBD tube was removed accidentally on the fourth hospital day. At first, the pain related to PTBD tube was alleviated. However, the afferent loop syndrome aggravated on the sixth hospital day, and the patient complained of abdominal pain more frequently on that day. The LSTM-based model underestimates the frequency of CPE compared to real-world pain records. The performance of LSTM-based model during hospitalization was MCC of 0.0467, AUROC of 0.4691, and AUPRC of 0.1379.

## Discussion

In this study, we explored the feasibility of deep learning methods to predict the onset time of CPE in cancer patients at the time of hospitalization. The NRS pain records showed circadian patterns and correlated with NRS pain patterns of the previous days. In particular, the NRS scores were positively correlated with the closeness from the forecast date and the size of time binning. The LSTM-based model showed a good performance by achieving the best performance in the experiments with 24 h input length and 1-h time bin (MCC: 0.1721). The performance was improved in the experiments with more extended input data and larger binning size, which showed the best performance in the 120 h input length and 12 h bin lengths (MCC: 0.4927). Considering this model performance was significantly better than the base model performance (Table 1), our study showed that the NRS pain could be predictable using deep learning-based models.

The NRS pain records showed circadian pain patterns, mostly recorded during 8:00–10:00 h and 16:00–18:00 h, near the rounding time (Fig. 3A). All recorded pain episodes were pre-processed in 24-h increments to make the data reflect this circadian pattern. Zero padding was performed to set the start and end of all CPE episodes to 0:00 h. After the pre-processing, the performance was significantly improved (Supplementary Table 3). Meanwhile, the input record that was obtained one day before the prediction had the highest correlation and similarity with the forecast period when compared to the records on other days (Fig. 3B). This pain pattern may be affected by pain management in the hospital, including elevation of dose or frequency of opioids and other interventions. This data characteristic could be the reason why unidirectional RNN model, including LSTM and GRU, showed competitive performance compared to others, although the unidirectional RNN model is well known to be vulnerable in the case of long-term dependence. Meanwhile, as described in Supplementary Figure 1B, pain patterns could be related to various factors, including acute events and treatment patterns. As current models could not predict pain patterns reflecting this acute change, time series data reflecting these factors could make the model perform better.

Pérez-Hernández et al. investigated breakthrough pain characteristics and patterns using the Alberta breakthrough pain assessment tool^25^. This study showed that 42.6% of patients could correctly predict the occurrence of breakthrough pain. Another 20.5% of patients could accurately estimate breakthrough pain on certain occasions. This is mostly because CPE is associated with pose or movement of patients. In an earlier study, as per the answers of 81.5% of the patients, the duration of onset time to the peak intensity of breakthrough pain was less than 30 min. Considering interval breakthrough pain is twice as much as onset to peak time, we first tried to make a predictive model inputting the serial pain log data divided by an hour. However, as we used the zero-input method for missing values, the data had high sparsity after the 1-h binning (CPE: 6.0% of the total dataset). Therefore, we performed ablation studies with larger intervals binning up to 12 h, which was the least clinically available, and the sparsity of CPE was improved (CPE: 44.5% of the total dataset) (Supplementary Table 2). Meanwhile, the length of the input record also affected the model performance. However, the extent was less significant compared to the change of time binning length. It is necessary to reflect on these data characteristics and optimize them according to the clinical setting.

Our study has certain limitations. The most prominent limitation is its single center-based design, which might limit the generalizability of our data. In addition, we used NRS records divided by hours and simply defined the breakthrough pain as the time interval with the records with an NRS above 4. By following this protocol, we have excluded many other characteristics of cancer pain, that might limit the interpretation. Additionally, our study investigated univariable models with simple structures as our goal was to explore the feasibility of pain patterns. Nevertheless, our models showed adequate performance even though there were few input data types. Subsequent validation studies, including detailed data and sophisticated model structure, would make the model perform better and more applicable.

In conclusion, our study showed that cancer pain could be predictive using deep learning models. Though our exploratory study has limitations, further research could improve the model performance, and verification study could make our model applicable in real-world practice.

## Supplementary Information

Supplementary Information 1.

Supplementary Information 2.

## Data Availability

The data that support the findings of this study are available from the institutional review board of Samsung Medical Center, while restrictions apply to the availability of these data that were used under license for the current study and so are not publicly available. However, data are available from the corresponding author upon reasonable request and with the permission of the institutional review board of Samsung Medical Center.

## Code availability

Scripts for pre-processing, training, and evaluation in this work were written in Python. They are available with accompanying documentation at https://github.com/YOONHO-CHOI/BTcP-prediction.
