# Supplementary material for: Clinical relevance of deep learning models in predicting the onset timing of cancer pain exacerbation
Source: Sci Rep. 2023 Jul 17;13:11501. doi: 10.1038/s41598-023-37742-5 (PMC10352236; doi:10.1038/s41598-023-37742-5)
Supplement: Supplementary file 1 — Supplementary Information 1. [file 41598_2023_37742_MOESM1_ESM.docx]

**Supplementary Table 1. Baseline characteristics**

| **Variables** | **Total cohort**  **(N=3,431)** | **Total admission**  **(N=4,870)** | **Hospitalization days** | **Frequency of NRS records per day** | **Frequency of BTcP per day** |
| --- | --- | --- | --- | --- | --- |
| **Total** |  |  | 14.96 (5.46-195.40) | 2.74 (0.17-12.22) | 1.01 (0-1.31) |
| **Age (years)** | 58 (15-89) | 57 (15-89) |  |  |  |
| ≥ 60 years | 1,338 (39.0%) | 1,738 (35.7%) | 13.83 (5.46-195.42) | 2.63 (0.19-2.91) | 0.91 (0-6.91) |
| < 60 years | 2,093 (61.0%) | 3,132 (64.3%) | 15.92 (5.58-194.92) | 2.82 (0.17-12.22) | 1.07 (0-7.85) |
| **Sex** |  |  |  |  |  |
| Male | 2,047 (59.7%) | 2,941 (60.4%) | 14.96 (5.46-195.42) | 2.76 (0.17-12.22) | 1.02 (0-7.85) |
| Female | 1,384 (40.3%) | 1,929 (39.6%) | 14.96 (5.58-194.92) | 2.72 (0.19-9.76) | 1.0 (0-6.91) |
| **Types of cancer** |  |  |  |  |  |
| Aplastic anemia | 18 (0.5%) | 20 (0.4%) | 29.50 (7.79-93.10) | 2.41 (0.83-3.72) | 1.01 (0-2.46) |
| Acute myeloid leukemia | 249 (7.3%) | 491 (10.1%) | 28.00 (5.75-179.17) | 1.78 (0.31-9.25) | 0.67 (0-0.87) |
| Acute lymphoid leukemia | 36 (1.0%) | 90 (1.8%) | 28.85 (5.83-147.91) | 2.18 (0.58-6.43) | 1.23 (0.07-3.98) |
| Chronic myeloid leukemia | 14 (0.4%) | 25 (0.5%) | 27.38 (5.96-74.58) | 1.96 (0.32-6.51) | 0.81 (0-5.08) |
| MDS* & MPD* | 120 (3.5%) | 157 (3.2%) | 21.85 (5.58-176.71) | 2.02 (0.17-11.83) | 0.78 (0-6.22) |
| Lymphoma | 491 (14.3%) | 746 (15.3%) | 17.00 (5.67-195.42) | 2.25 (0.24-5.53) | 0.80 (0-4.02) |
| Plasma cell neoplasm | 219 (6.4%) | 284 (5.8%) | 26.81 (6.79-135.92) | 2.05 (0.21–6.63) | 0.90 (0.05-3.79) |
| Pancreaticobiliary cancer | 137 (4.0%) | 171 (3.5%) | 10.92 (5.46-66.13) | 3.49 (0.55-9.70) | 1.37 (0.03-6.33) |
| Stomach & esophageal cancer | 380 (11.1%) | 552 (11.3%) | 10.92 (5.63-80.13) | 3.50 (0.56-10.01) | 1.44 (0-6.74) |
| Genitourinary cancer | 221 (6.4%) | 328 (6.7%) | 13.77 (5.67-78.79) | 2.97 (0.49-8.48) | 1.02 (0-5.11) |
| Colorectal cancer & small bowel cancer | 201 (5.9%) | 258 (5.3%) | 11.25 (5.63-58.75) | 3.48 (0.41-9.74) | 1.29 (0-6.91) |
| Breast cancer | 223 (6.5%) | 304 (6.2%) | 12.67 (5.71-103.38) | 3.03 (0.39-9.73) | 0.91 (0-6.20) |
| Lung cancer | 745 (21.7%) | 922 (18.9%) | 11.00 (5.54-109.08) | 1.13 (0-6.13) | 0.50 (0.06-2.21) |
| Sarcoma | 74 (2.2%) | 96 (2.0%) | 11.79 (5.58-44.96) | 3.03 (0.78-7.61) | 1.19 (0-4.68) |
| Skin cancer & melanoma | 41 (1.2%) | 62 (1.3%) | 10.40 (5.67-52.58) | 3.36 (1.6-9.02) | 1.01 (0-5.65) |
| Liver cancer | 58 (1.7%) | 69 (1.4%) | 9.38 (5.63-42.71) | 3.41 (1.40-6.98) | 1.40 (0.05-3.94) |
| Head & neck cancer | 92 (2.7%) | 145 (3.0%) | 9.81 (5.6-78.58) | 3.76 (0.47-12.22) | 1.57 (0-7.85) |
| Others | 112 (3.3%) | 150 (3.1%) | 10.79 (5.71-95.91) | 3.61 (0.55-9.43) | 1.54 (0-6.47) |

*MDS: myelodyplastic syndrome; MPD: myeloproliferative disease

Data are presented as no. (%) or median (range)

| **Supplementary Table 2. Pain records according to time-bin length** | | | |
| --- | --- | --- | --- |
| **Variables**  **Time-bin**  **length (**$\boldsymbol{\tau}$**)** | **Total time binned pain records** | **Records of BTcP** | **BTcP per total records** |
| **1** | 1,311,240 | 78,376 | 6.0% |
| **2** | 655,620 | 75,079 | 11.5% |
| **3** | 437,080 | 72,858 | 16.7% |
| **4** | 327,810 | 70,244 | 21.4% |
| **6** | 218,540 | 64,032 | 29.3% |
| **8** | 163,905 | 58,117 | 35.5% |
| **12** | 109,270 | 48,663 | 44.5% |

Data are presented as no. (%) or median (range)

| **Supplementary Table 3. Comparison of performance before and after transformation** | | | | | | | | | |
| --- | --- | --- | --- | --- | --- | --- | --- | --- | --- |
| **Input length**  **Time-bin**  **length (**$\boldsymbol{\tau}$**)** | **24 h** | | | **72 h** | | | **120 h** | | |
|  | **Before transformation** | **After transformation** | | **Before transformation** | **After transformation** | | **Before transformation** | | **After transformation** |
| **1** | 0.1686 | | 0.1721 | 0.1800 | | 0.1889 | 0.1791 | 0.1861 | |
| **2** | 0.2363 | | 0.2417 | 0.2512 | | 0.2650 | 0.2511 | 0.2635 | |
| **3** | 0.2816 | | 0.2900 | 0.3046 | | 0.3238 | 0.3069 | 0.3231 | |
| **4** | 0.3303 | | 0.3365 | 0.3547 | | 0.3722 | 0.3457 | 0.3771 | |
| **6** | 0.3725 | | 0.3718 | 0.4115 | | 0.4233 | 0.4003 | 0.4278 | |
| **8** | 0.4055 | | 0.4074 | 0.4434 | | 0.4624 | 0.4488 | 0.4730 | |
| **12** | 0.4180 | | 0.4179 | 0.4665 | | 0.4172 | 0.4732 | 0.4927 | |

The performance was evaluated based on the Matthew correlation coefficient (MCC)

**Supplementary Table 4. Comparison of performance according to the cancer types in the 120-hour of input length and 12-hour time bin LSTM based model**

| **Type of cancers** | MCC | ROC-AUC | PR-AUC |
| --- | --- | --- | --- |
| Aplastic anemia | 0.663 | 0.825 | 0.845 |
| Acute myeloid leukemia | 0.520 | 0.825 | 0.724 |
| Acute lymphoid leukemia | 0.499 | 0.815 | 0.789 |
| Chronic myeloid leukemia | 0.432 | 0.835 | 0.651 |
| MDS* & MPD* | 0.496 | 0.818 | 0.737 |
| Lymphoma | 0.478 | 0.801 | 0.710 |
| Plasma cell neoplasm | 0.406 | 0.772 | 0.699 |
| Pancreaticobiliary cancer | 0.480 | 0.810 | 0.786 |
| Stomach & esophageal cancer | 0.406 | 0.793 | 0.715 |
| Genitourinary cancer | 0.456 | 0.806 | 0.717 |
| Colorectal cancer & small bowel cancer | 0.589 | 0.831 | 0.797 |
| Breast cancer | 0.481 | 0.807 | 0.718 |
| Lung cancer | 0.477 | 0.808 | 0.705 |
| Sarcoma | 0.398 | 0.773 | 0.709 |
| Skin cancer & melanoma | 0.316 | 0.803 | 0.776 |
| Liver cancer | 0.294 | 0.741 | 0.565 |
| Head & neck cancer | 0.594 | 0.830 | 0.815 |
| Others | 0.452 | 0.797 | 0.685 |

*MDS: myelodyplastic syndrome; MPD: myeloproliferative disease
