# Supplementary material for: Clinical relevance of deep learning models in predicting the onset timing of cancer pain exacerbation
Source: Sci Rep. 2023 Jul 17;13:11501. doi: 10.1038/s41598-023-37742-5 (PMC10352236; doi:10.1038/s41598-023-37742-5)
Supplement: Supplementary file 2 — Supplementary Information 2. [file 41598_2023_37742_MOESM2_ESM.pdf]

Supplementary Figure 1.

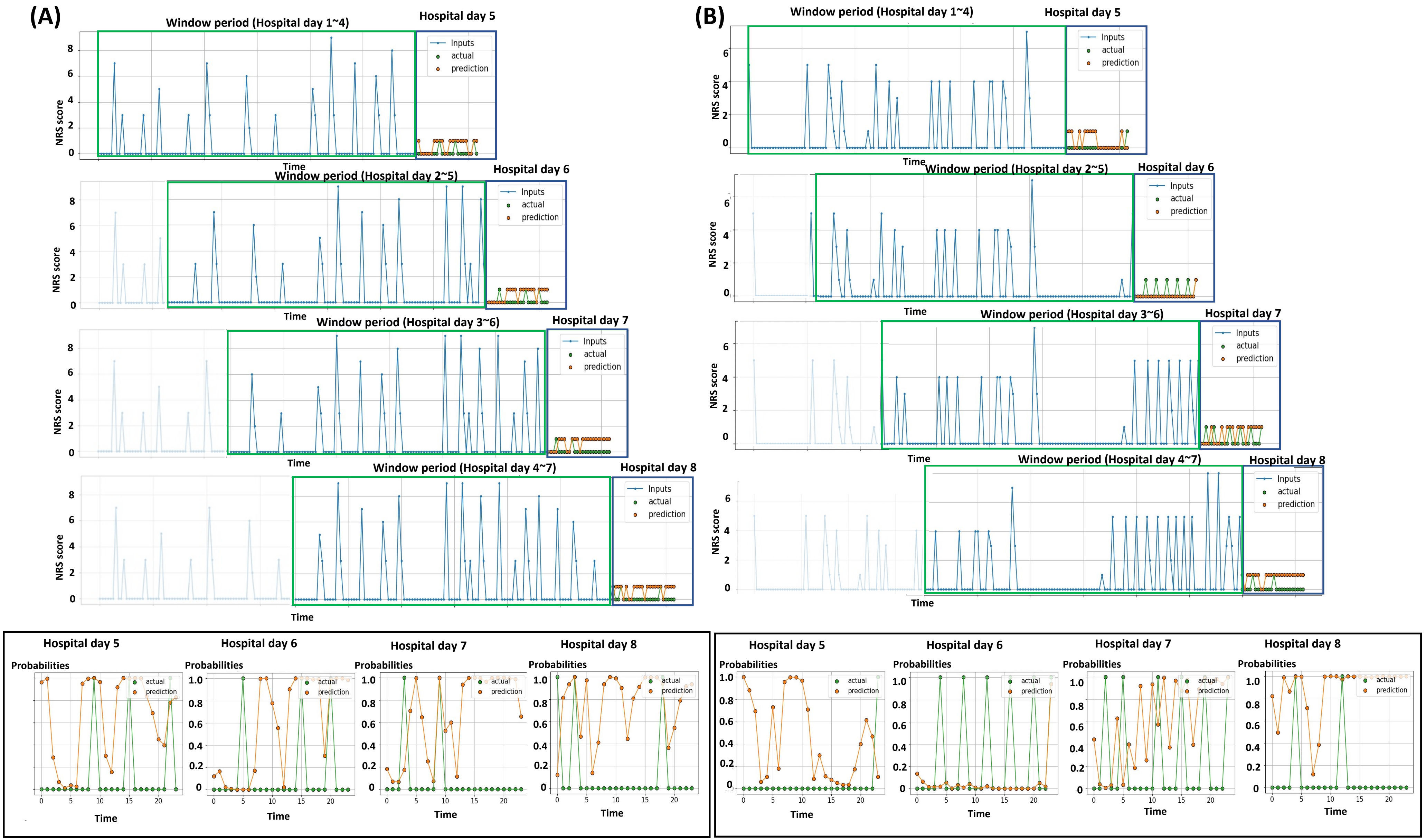

Representative cases for predicting for onset time of cancer pain exacerbation using serial pain records derived from patients.

(a) The case of the patient with renal cell carcinoma who complained of back pain

(b) The case of the stomach cancer patient who underwent PTBD and L-tube insertion
